# Store‐operated calcium entry mediates hyperalgesic responses during neuropathy

**DOI:** 10.1002/2211-5463.13699

**Published:** 2023-08-28

**Authors:** Wei Wang, Qiru Wang, Jinlu Huang, Hong Li, Fangjie Li, Xue Li, Ruimei Liu, Ming Xu, Jinghong Chen, Yemeng Mao, Le Ma

**Affiliations:** ^1^ Shanghai Mental Health Center Shanghai Jiao Tong University School of Medicine Shanghai China; ^2^ Shanghai Key Laboratory of Psychotic Disorders China; ^3^ Department of Pharmacy Fudan University Shanghai Cancer Center, Minhang Branch Shanghai China; ^4^ Department of Pharmacy Shanghai Jiao Tong University Affiliated Sixth People's Hospital China; ^5^ Department of Laboratory Medicine Changhai Hospital Shanghai China; ^6^ Department of Digital and Cosmetic Dentistry, School & Hospital of Stomatology Tongji University Shanghai China

**Keywords:** neuronal activity, neuropathy, SKF96365, SOCE, store‐operated calcium entry, YM‐58483

## Abstract

Neuropathic pain (NP), resulting from nerve injury, alters neural plasticity in spinal cord and brain via the release of inflammatory mediators. The remodeling of store‐operated calcium entry (SOCE) involves the refilling of calcium in the endoplasmic reticulum via STIM1 and Orai1 proteins and is crucial for maintaining neural plasticity and neurotransmitter release. The mechanism underlying SOCE‐mediated NP remains largely unknown. In this study, we found SOCE‐mediated calcium refilling was significantly higher during neuropathic pain, and the major component Orai1 was specifically co‐localized with neuronal markers. Intrathecal injection of SOCE antagonist SKF96365 remarkably alleviated nerve injury‐ and formalin‐induced pain and suppressed c‐Fos expression in response to innocuous mechanical stimulation. RNA sequencing revealed that SKF96365 altered the expression of spinal transcription factors, including Fos, Junb, and Socs3, during neuropathic pain. In order to identify the genes critical for SKF96365‐induced effects, we performed weighted gene co‐expression network analysis (WGCNA) to identify the genes most correlated with paw withdrawal latency phenotypes. Of the 16 modules, MEsalmon module was the most highly correlated with SKF96365 induced effects. Kyoto Encyclopedia of Genes and Genomes (KEGG) analysis showed that the enriched genes of MEsalmon module were significantly related to Toll‐like receptor signaling, steroid biosynthesis, and chemokine signaling, which may mediate the analgesic effect caused by SKF9636 treatment. Additionally, the SOCE antagonist YM‐58483 produced similar analgesic effects in nerve injury‐ and formalin‐induced pain. Our results suggest that manipulation of spinal SOCE signaling might be a promising target for pain relief by regulating neurotransmitter production and spinal transcription factor expression.

## Graphical abstract

Inflammatory factors promote microglial activation and neuronal transmission via sensitized SOCE and calcium influx in synapses, resulting in activation of nocifensive reflexes in spinal cord. SKF96365 can suppress SOCE‐mediated calcium influx and neuronal transmitter release, which subsequently alleviates cellular signaling defects, including Toll‐like receptor signaling, steroid biosynthesis, chemokine signaling, and c‐Fos expression, as well as glutamatergic transmission in neuropathic rats.

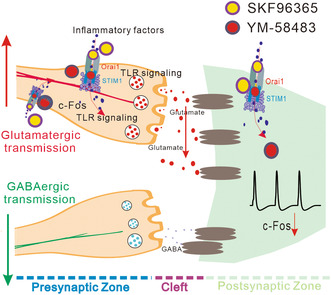

**Abbreviations**
ACSF: artificial cerebrospinal fluid
ANOVA: analysis of variance
CNS: central nervous system
DRG: dorsal root ganglion
GFAP: glial fibrillary acidic protein
GO analysis: gene ontology analysis
GPCRs: G‐protein‐coupled receptors
Iba1: Ionized calcium binding adaptor molecule 1
KEGG: Kyoto Encyclopedia of Genes and Genomes
NeuN: neuronal nuclei
NP: neuropathic pain
SNL: spinal nerve ligation
SOCE: store‐operated calcium entry
STIM1: stromal interaction molecule 1
TBST: triple‐phase buffered saline
WGCNA: weighted gene co‐expression network analysis

Neuron expressed G‐protein‐coupled receptors (GPCRs) are crucial for intracellular calcium mobilizations coupling with a variety of receptors or ion channels. This cross‐talk between GPCRs and ion channels, being responsible for neuronal transmitters and neuronal activity in pain states, may have a reciprocal implication for inflammatory risks during pathological process. As proved in rodents, the subunits of adenosine receptor antagonists remarkably potentiated mechanical anti‐allodynia effects in pain rats by regulating transient receptor potential vanilloid 1 (TRPV1) channel‐mediated intracellular calcium intensity, indicating that GPCRs mediated control of calcium dynamics [1]. In addition, cannabinoid agonists WIN55212 produced better antinociceptive activity in rats when combining with μ‐opioids agonism tramadol [2]. Thus, GPCRs regulated receptors relationships that provided a broader overview in pain management.

Store‐operated calcium (Ca^2+^) entry (SOCE) is the main calcium channel responsible for intracellular calcium homeostasis, a process regulated by GPCR‐stimulated diacylglycerol (DAG) production [3]. Research has linked SOCE to neuronal plasticity and psychiatric disorders, suggesting that it plays a dynamic role in tissue repair and contributes to the maintenance of dendritic spines, responsible for transmitter release and synaptic plasticity [4]. SOCE is activated when Ca^2+^ stores are depleted, by activating membrane Orai1 coupled with endoplasmic Ca^2+^ sensor stromal interaction molecule 1 (STIM1) [5]. The molecular components of SOCE may provide new insights into different physiological and pathological states of the central nervous system (CNS), including neuronal activity, autophagy, and apoptosis [6], and increase the risk of nocifensive reflexes during NP [7].

Sustained increases of intracellular calcium cause the nervous system to become uniquely sensitive to changes in synaptic transmission, which may have important implications for pathological development and pain [8]. The interaction between STIM1 and Orai1 is originally activated in ER stress, oxidative stress, hypoxia, mitochondrial damage, and nerve injury after changes in intracellular calcium levels [9]. Altered STIM1 leads to truncated SOCE, resulting in a reduction in neuronal calcium homeostasis, as well as the sensitivity of sensory neurons and their intrinsic firing properties, which might play a critical role in pain‐induced hyperexcitability of sensory neurons [10, 11]. Nerve injury can significantly increase synaptic strength by regulating calcium channels, specifically SOCE [12]. SOCE exerts a strong membrane depolarization and regulates the action potential of neurons in the spinal dorsal horn, subsequently contributing to the promotion of depolarization and neuronal excitability in response to nocifensive reflexes [13].A pharmacological blockade of SOCE function in CA1 significantly abolishes the accumulation of Ca^2+^ and glutamatergic transmission [14]. Thus, calcium entry often used as a target for the treatment of various mental disorders.

Store‐operated calcium entry maintains intrinsic excitability and modulates neuronal calcium signaling, and its deficiency is remarkably associated with major developmental impairments in a rodent pain model [15]. Orai1 and STIM1 collaborate together and functionally alter calcium influx in dorsal root ganglion (DRG), transforming nociceptive information, particularly in a spared nerve injury (SNI) model [16]. Inflammatory mediators induce behavioral sensitization, which disrupts the conjunctions of STIM1‐Orai1 tethering, significantly decreases intracellular Ca^2+^ levels in primary neuron culture, and diminishes nocifensive responses in rodents [17]. Genetically targeting Orai1 has a distinct effect on pain sensations [18]. Pharmacological disruptions of STIM1 and Orai1 suppress Ca^2+^ influx, exerting a strong anti‐allodynic effect on both complete Freund's adjuvant (CFA)‐ and formalin‐induced nocifensive behavior [19, 20]. By stimulating the expression of STIM1 and Oria1, moxibustion has a similar anti‐inflammatory effect on the production of TNF‐α, IL‐1β, and PGE‐2 during the early stage of pain [19, 21, 22]. In addition, junctophilin‐4 (JPH4) facilitates STIM1‐Orai1 joint formation. Knockdown of JPH4 results in the ablation of ER Ca2^+^ store refill in sensory neurons, which leads to inflammatory‐mediated behavioral abnormalities in mice [23]. These findings suggest that SOCE activity in neurons may drive the progression of pain by disrupting cell signal adaptions to painful sensations [12, 24]. However, the mechanism for SOCE‐mediated behavioral sensitization, including neuronal plasticity and subsequent gene expression during neuropathic pain, remains largely unknown.

The present study identified a significant increase in SOCE‐mediated calcium influx following injury‐induced neuropathic pain. Pharmacologically inhibition of SOCE by both SKF96365 and YM‐58483 had an analgesic effect on NP. Furthermore, SKF96365 treatment rescued neuronal c‐Fos^+^ expression in neuropathic rats. Electrophysiological recordings demonstrated that SOCE inhibition was crucially involved in spinal synaptic plasticity during pain. RNA sequencing was utilized to evaluate gene expression associated with neuropathic pain before and after blocking STIM1‐Orai1 tethering in the spinal dorsal horn. SKF96365 specifically rescued the phenotypes that related to cytokine activity and cellular responses to lipopolysaccharide and IL‐1β following spinal nerve injury in the dorsal spinal cord. Weighted gene co‐expression network analysis (WGCNA) identified critical salmon modules that differed between the SOCE‐mediated pathways associated with neuropathic pain and Toll‐like receptor signaling, steroid biosynthesis, and chemokine signaling pathways. We also showed that the anti‐allodynia phenotypes induced by SKF96365 and YM‐58483 correlated with spinal inflammatory responses during neuropathic pain. Furthermore, YM58483 showed almost similar analgesic effects compared to SKF96365. To summarize, the findings of this study revealed that SKF96365 and YM58483 produced antinociception in neuropathic rats after pharmacologically blocking SOCE in the spinal cord.

## Materials and methods

### Drugs and reagents

SKF96365 and YM‐58483 were obtained from Sigma‐Aldrich (St Louis, MO, USA) and Mykelin (Shanghai, China). SKF96365 and YM‐58483 were applied and delivered as discussion previously [20, 25].

### Animals

Adult male Wistar rats were acquired from the Shanghai Experimental Animal Institute for Biological Sciences (Shanghai, China) and were housed in a temperature‐controlled environment at 23 ± 2 °C, with humidity ranging between 25% and 70%, and with a 12/12‐h light/dark cycle. The rats had access to food and water *ad libitum*, were carefully handled, and were allowed to adapt for a week before the start of the experiments. The procedures and protocols were approved by the Animal Care and Welfare Committee of the Shanghai Jiao Tong University (Shanghai, China, A2018081), and the experiments were performed following the animal care guidelines of the National Institutes of Health (NIH).

### Spinal nerve ligation (SNL) and behavior assessment

The rats were anesthetized using an intraperitoneal injection of 50 mg·kg^−1^ sodium pentobarbital and the L5 and L6 vertebrae were gently dissected as described previously [26]. In summary, the left sides of L5 and L6 were gently isolated using a glass rod and then tightly ligated with a 6–0 silk thread. After suturing the wounds, the rats were allowed to recover for a week before conducting the behavior test. Each rat was individually acclimatized to the atmosphere in the transparent plexiglass boxes for at least 30 min before measuring the hind paw threshold. An electronic Von Frey hair (IITC Life Science Inc., Woodland Hill, CA, USA) was used to stimulate the paw non‐invasively, with a range of 0.1–90 g. The withdrawal of the paw was measured, and an average of three measurements was calculated for each point. Rats with a paw withdrawal threshold of < 8 g were put into the SNL group, which involved spinal nerve ligations. The sham group was set up in the same way but without any ligation of L5 and L6.

### Immunofluorescence staining

The rats were anesthetized with 50 mg·kg^−1^ sodium pentobarbital, followed by a perfusion with 30 mL 0.9% saline and 100 mL paraformaldehyde solutions. Spinal enlargements of L3–L5 were fixed in 4% paraformaldehyde solutions overnight and dehydrated twice in 30% sucrose at 4 °C until precipitation. Tissue was cut into 30 μm slices and incubated with 10% goat serum (v/v) and 0.5% Triton X‐100 (v/v) in PBS at RT for 2 h. The slices were incubated with the primary antibodies, c‐Fos (1 : 300), Orai1 (1 : 300), NeuN (1 : 300), GFAP (1 : 300), and Iba1 (1 : 300), at 4 °C for 24 h, and subsequently incubated with secondary antibody at RT for 2 h. Images were visualized using a laser‐scanning confocal microscope (Olympus,Tokyo, Japan).

### Rat formalin test

The formalin test was performed as previous study [27]. Briefly, rats were injected with 50% formalin subcutaneously on the left side of the hindpaw. The rats were immediately transferred to the transparent polycarbonate boxes. Nocifensive behavior was assessed by counting the number of the formalin‐injected paw flinches within 1 min at 0, 10, 20, 30, 40, 50, 60, 70, 80, and 90.

### Western blot

Ipsilateral spinal enlargement of L3‐L5 were dissected by anesthetizing with 50 mg·kg^−1^ sodium pentobarbital. 200 μL of RIPA lysis buffer containing protease and phosphatase inhibitors was used. After an ice bath for 15 min, the proteins were separated by centrifugation at 12 000 ***g*** and 4 °C for 20 min. The protein concentration of the samples was determined using a BCA protein assay kit. Samples containing 30 μg of protein were separated on a sodium dodecyl sulfate‐polyacrylamide gel and transferred to a polyvinylidene difluoride membrane. The membranes were blocked with 5% skimmed milk and tween‐containing triple‐phase buffered saline (TBST) for 2 h at room temperature. The membranes then incubated with primary antibodies at 4 °C overnight. All primary antibodies were diluted in TBST with 5% skimmed milk. The membranes were then washed with TBST and incubated with the corresponding horseradish peroxidase‐conjugated secondary antibodies for 2 h at room temperature. Signals were detected with enhanced chemiluminescence reagents. Results were analyzed and quantified by Image J.Intrathecal catheterization and injection.

A PE‐10 catheter (outer diameter 0.55 mm, inner diameter 0.3 mm) was inserted into lumber spinal under anesthesia as described previously [28]. A catheter was tightly anchored in the subcutaneous tissue. The position was verified using an intrathecal injection of 10 μL 4% lidocaine following a flush with 15 μL ACSF. The injected rats exhibited instant bilateral hindlimb paralysis. The drug was delivered in 10 μL artificial cerebrospinal fluid by intrathecal injection following a 15 μL flush.

### Spinal cord slice preparations

The spinal L3–L5 segments were quickly dissected as described previously. In brief, the spinal enlargements were immersed in ice‐cold cutting solution for 90 s and 300 μm slices were sectioned with a VT1200s vibratome (Leica Biosystems, Vista, CA, USA) and placed in cutting buffer. The spinal slices were incubated in oxygenated ACSF to recover at 32 °C for 30 min and transferred to room temperature before making the electrophysiological recordings.

### Electrophysiological recordings

Spinal slices of the L3‐L5 segments were continuously perfused with ACSF in a recording chamber. To record the I_CRAC_ currents, nimodipine and spermine chloride were used to block the L‐type calcium channel and Mg^2+^‐mediated currents. Tetraethylammonium and tetrodotoxin were added to the external solution to block tetraethylammonium‐sensitive K^+^ and Na^+^ currents using Cs‐based intervals (140 mm Cs‐gluconate, 10 mm Hepes, 1.1 mm EGTA, 2 mm MgCl_2_, 3 mm MgATP and 0.3 mm Tris–guanosine triphosphate, pH 7.4 adjusted with CsOH). I_CRAC_ and store‐operated calcium currents were recorded using a 100‐ms voltage ramp protocol (from +90 to −120 mV) from a −15 mV holding potential every 10 s. The I_CRAC_ magnitude was measured at −120 mV for analysis. Thus, I_CRAC_ was normalized to the membrane capacitance to exclude possible changes in cell size measured by pA/pF.

### RNA isolation, library preparation, and sequencing

Total spinal mRNA of the enlargement region was extracted using TRIzol. An OD ratio of 1.8–2.1 was used for the following experiments as previously [29]. A RefertAid First Strand cDNA synthesis kit (Thermo scientific, Waltham, MA, USA) was used to produce cDNA on oligo (dT) magnetic beads. PCR was used to create a sequencing library on Illumina novaseq™ 6000 (Illumina, Inc., San Diego, CA, USA) with a sequencing format of PE150.

### RNA‐Seq data and phenotype characteristics of rat from WGCNA

Raw data were transformed into gene symbols using ftp://ftp.ensembl.org/pub/release‐101/fasta/rattus_norvegicus/dna/ and relative gene expression was generated. After producing the final transcriptome, stringTie and edgeR were used to calculate the expression of each transcript. StringTie was used to calculate individual mRNA expression in FPKM. Differentially expressed mRNAs and genes were selected using edgeR package, and a log_2_ (fold change) > 1 or log_2_ (fold change) < −1 was considered statistically significant (*P* < 0.05). For WGCNA analysis, the novel algorithm was used to determine relationship between gene expression personality and biological features [30]. In brief, wgcna r package (R Software Inc. San Francisco, USA) was used to calculate correlations and build an adjacency matrix based on pickSoftThreshold. Biological features were then transferred to the topological overlap matrix (TOM). The expression profiles were summarized and clustered in these modules relative to correlation coefficient.

### Statistics

All data analyses were performed by researchers who were blind to the treatment groups in our study. graphpad prism (version 7.0, GraphPad Software Inc., San Diego, CA, USA) was used to analyze the data. The results are shown as the mean ± SEM before coreldraw (2019 version, Corel Corporation, Ottawa, Canada) arrangement. The data were analyzed using one‐way ANOVA followed by Tukey's *post hoc* test. The criterion of statistical significance was set as *P* < 0.05.

## Results

### Nerve injury induced the overactivation of SOCE and I_Crac_ in the spinal dorsal horn

**Fig. 1:**
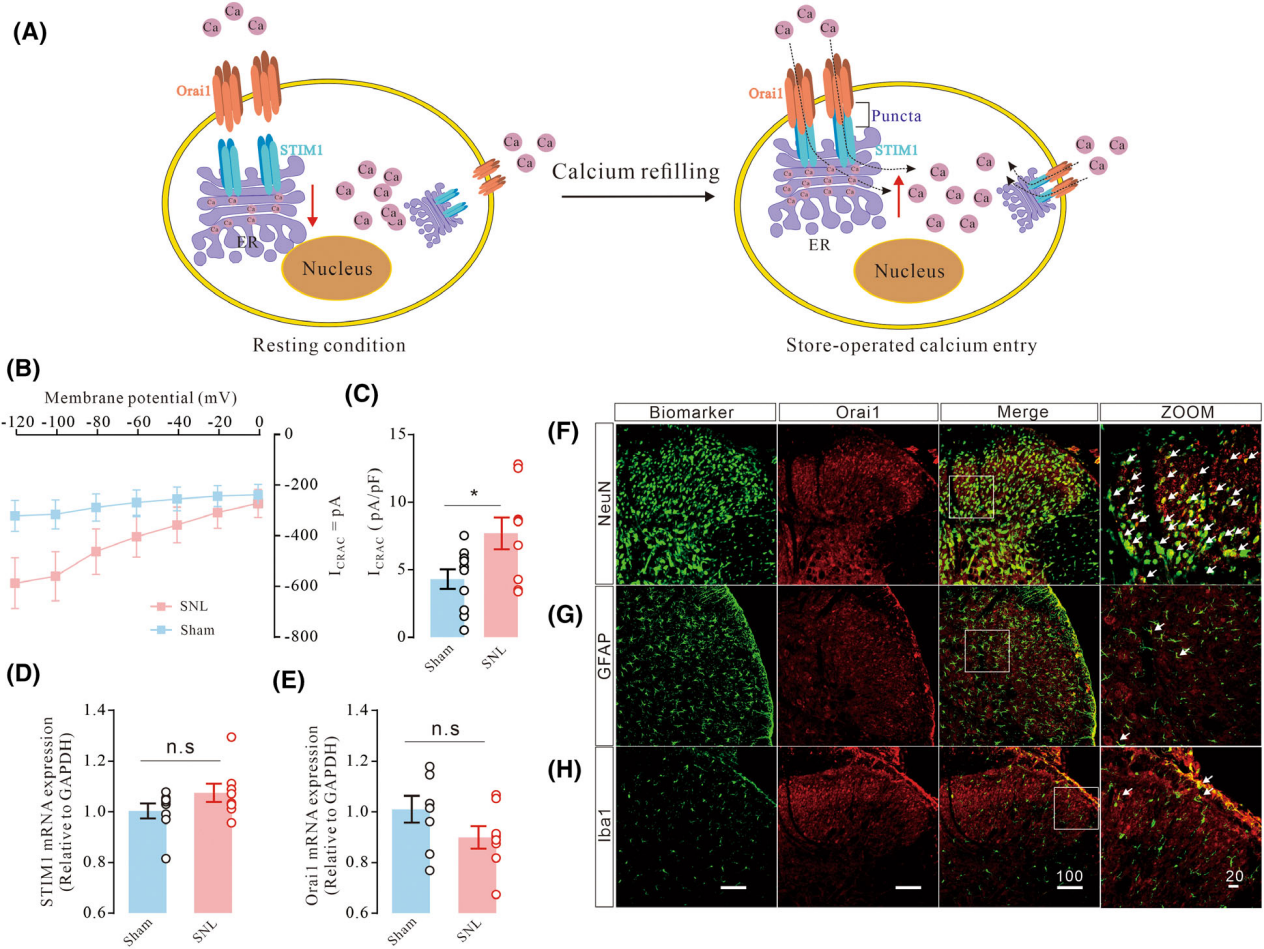
Diagram illustrated the process of store‐operated calcium entry (A). I–V relationship of I_CRAC_ measured in spinal neurons after nerve injury (B) and quantification of I_CRAC_ (B, C, *t*_17_ = 2.504, *P* = 0.0228, *P < 0.05, two‐tailed unpaired *t*‐test, mean ± SEM, *n* = 10) in neurons. qRT‐PCR analysis of STIM1 and Orai1 expression (D, E, STIM1, *t*_14_ = 1.540, *P* = 0.1459, Orai1, *t*_14_ = 1.605, *P* = 0.1309, two‐tailed unpaired *t* test, *n* = 8), Representative double‐staining immunofluorescence images of Orai1 (red) with NeuN (F, green), GFAP (G, green), and Iba1 (H, green). Scale bars: 100 and 20 μm, *n* = 3. Data are represented as mean ± SEM.

The association between STIM1 and Orai1 is physiologically important for maintaining neuronal activity and behavioral phenotypes (Fig. 1A). In current study, the voltage step protocol (hyperpolarized to −120 from +90 mV) showed that I_CRAC_ was increased after nerve injury (Fig. 1B,C, *t*_17_ = 2.504, *P* = 0.0228, *P < 0.05, two‐tailed unpaired *t*‐test, mean ± SEM, *n* = 10). In contrast, STIM1 and Orai1 mRNA expression in ipsilateral spinal enlargement of L3‐L5 remained unchanged after nerve injury (Fig. 1D,E, STIM1, *t*_14_ = 1.540, *P* = 0.1459, Orai1, *t*_14_ = 1.605, *P* = 0.1309, two‐tailed unpaired t test, mean ± SEM, *n* = 10). Since Orai1 is the major component required for calcium refilling, STIM1 and Orai1 expression is found in a wide range of cell types [31]. We found Orai1 strongly colocalized with NeuN, but showed lower levels of colocalization with GFAP and Iba1 (Fig. 1F–H, *n* = 3). Collectively, we demonstrated that the SOCE‐mediated calcium influx was remarkably increased followed nerve injury in neuropathic rats.

### SKF96365 alleviated mechanical allodynia during neuropathic pain

**Fig. 2:**
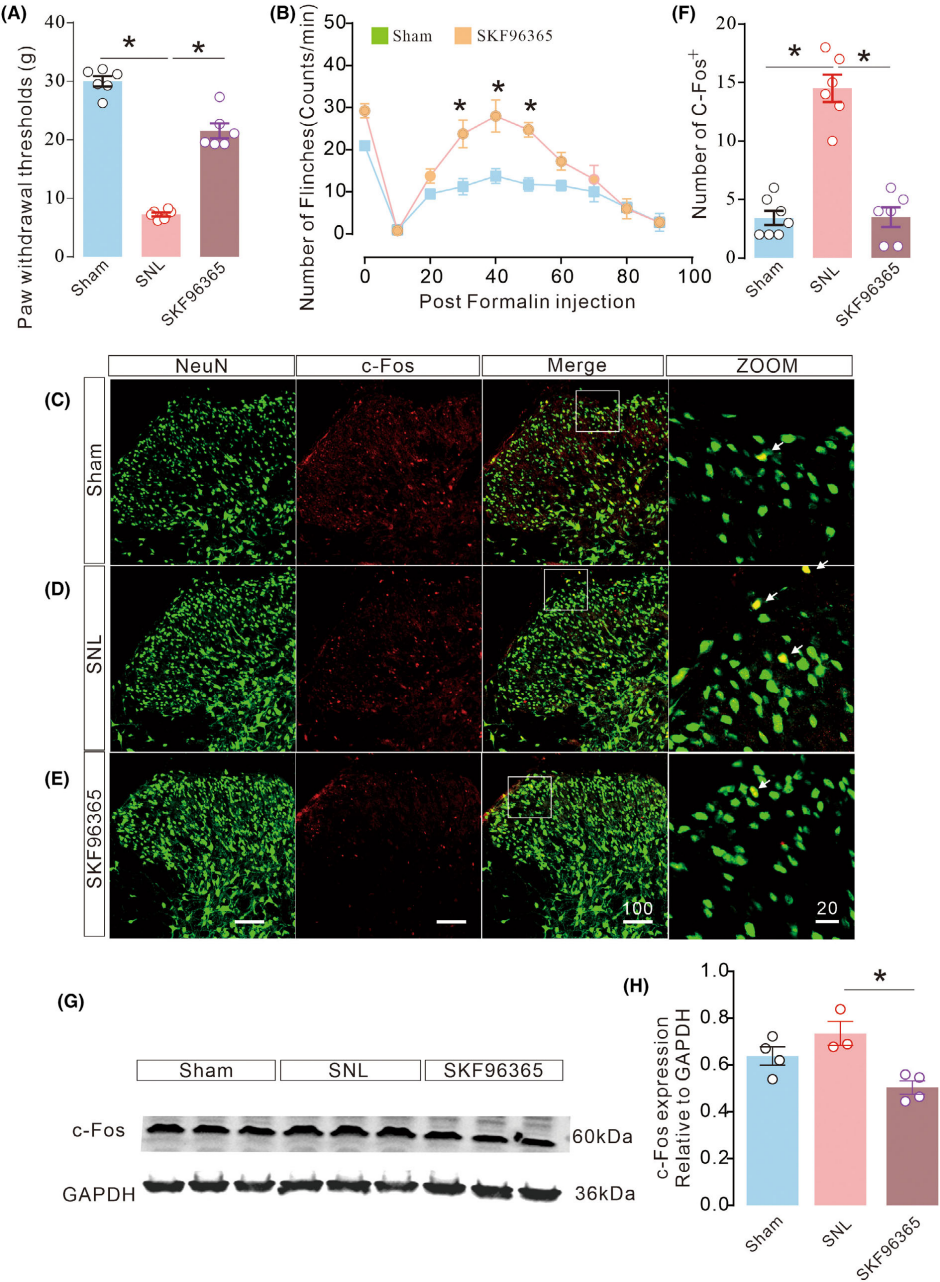
Attenuated pain responses in neuropathic rats following SKF96365 administrations. Administration of SKF96365 reduced mechanical allodynia (A, *t*_15_ = 157.7, *P* < 0.0001, *P < 0.05, two‐tailed unpaired *t*‐test; *n* = 6), Formalin pain (B, *F*_(9,60)_ = 50.84, *P* = 0.0004, *P < 0.05, two‐way ANOVA followed by Tukey's post‐tests, *n* = 4), and c‐Fos expression in IF (C–F, *F*_(2,17)_ = 42.82, *P* < 0.0001, *P < 0.05, one‐way ANOVA followed by Tukey's post‐tests, *n* = 6–7) and protein expression (G, H, *F*_(2,8)_ = 8.587, *P* = 0.0102, *P < 0.05, one‐way ANOVA followed by Tukey's post‐tests, *n* = 3–4) on the ipsilateral side toward noninvasive stimulus. Scale bars: 100 and 20 μm. Data are represented as mean ± SEM.

Rats with neuropathic pain often confer distinct susceptibility and exhibit abnormal neuronal activity toward several types of stimuli [32]. This study sought to examine the relationship between SOCE and neuronal activity during pain pathology following a noninvasive stimulus. Mechanical thresholds and c‐Fos expression in the spinal cord were assessed after SKF96365, a SOCE antagonist, treatments. SKF96364 shown to inhibit OCE‐mediated calcium refilling by 90–100% [33]. As expected, intrathecal SKF96365 treatment significantly alleviated hypersensitivity in SNL and formalin pain to 21.53 ± 1.282 g (Fig. 2A,B, *t*_15_ = 157.7, *P* < 0.0001, *P < 0.05, two‐tailed unpaired *t*‐test, *n* = 6; b, *F*_(9,60)_ = 50.84, *P* = 0.0004, *P < 0.05, two‐way ANOVA followed by Tukey's post‐tests, *n* = 4). c‐Fos protein is a biomarker of neuronal activity that can be used to measure pain circuits [34]. In this study, neuropathic rats exhibited higher levels of c‐Fos expression in the ipsilateral spinal dorsal horn than in the sham group (Fig. 1C), SKF96365 treatment markedly suppressed c‐Fos expression revealed by both immunofluorescent experiment (Fig. 2C–F, *F*_(2,17)_ = 42.82, *P* < 0.0001, *P < 0.05, one‐way ANOVA followed by Tukey's post‐tests, *n* = 6–7) and western blot experiment (Fig. 2G,H, *F*_(2,8)_ = 8.587, *P* = 0.0102, *P < 0.05, one‐way ANOVA followed by Tukey's post‐tests, *n* = 3–4). Thus, SKF96365 exerted a significant analgesic effect and alleviated neuronal circuitry activity in neuropathic pain by interrupting SOCE‐mediated calcium refilling.

### SKF96365 regulates gene expression profiles associated with analgesic responses

**Fig. 3:**
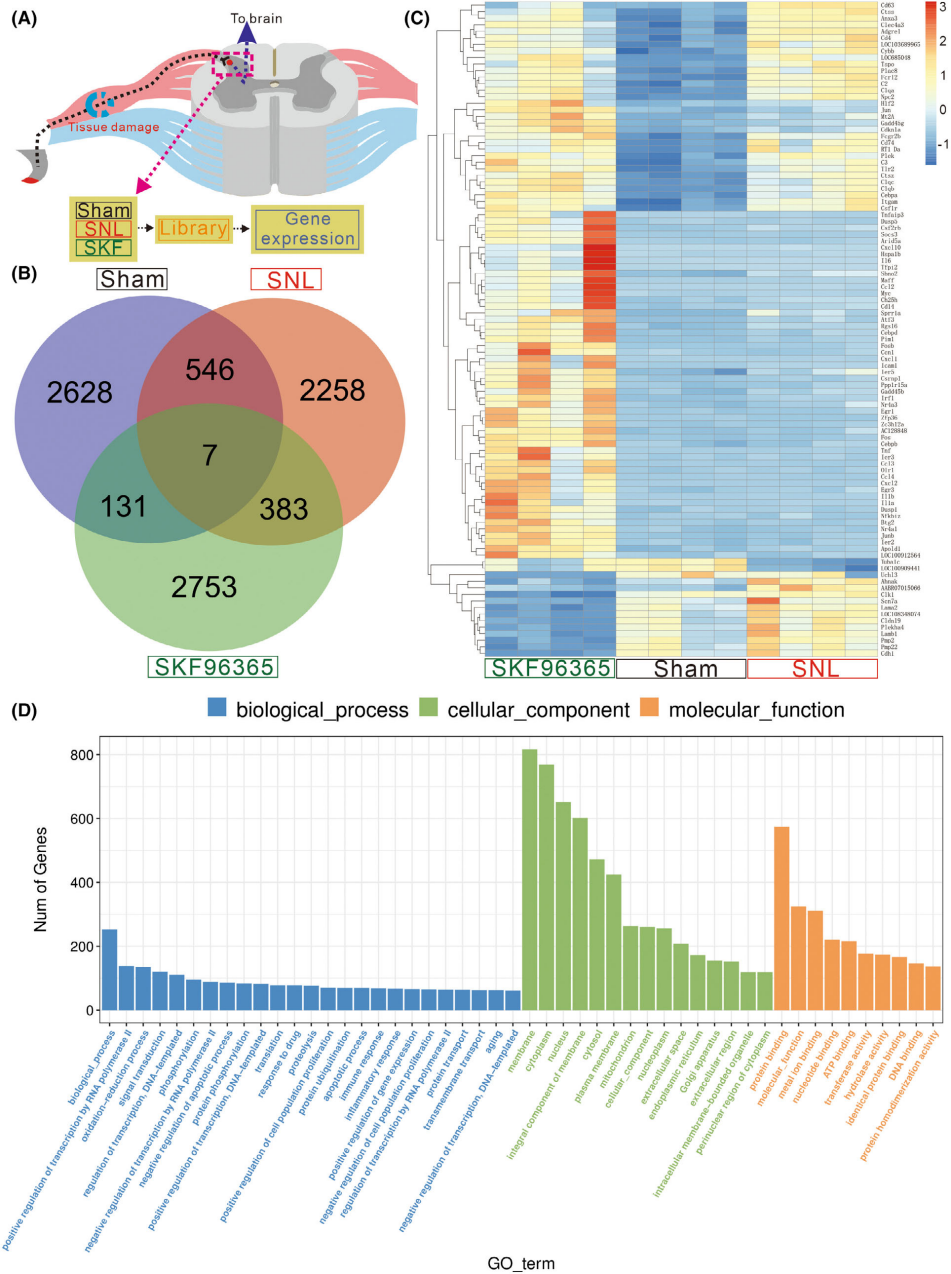
Transcriptome analyses suggest that inhibition of SOCE altered gene abnormities in pain responses. Schematic of the experimental timeline (A). Venn diagram of DEGs (SNL vs. Sham), DEGs (SKF96365 vs. SNL), and DEGs (Sham vs. SKF96365) (B). Heatmap of *z*‐score transformed normalized expression values for DEGs after SKF96365 treatment (C). Go enrichment analysis for the altered functions in relation to SKF96365 (D).

To better understand the SOCE signaling mechanism that occurs during neuropathic pain, transcriptome analyses were conducted following SKF96365 treatment (Fig. 3A). Differential gene expression between each group was assessed using the r deseq2 package (R Software Inc. San Francisco, USA). Several genes from different groups had statistically significant differences (Fig. 3B). A heatmap showed that SKF96365 treatment induced a strong reversal in the expression of these SNL‐associated genes, such as Fos, Junb, and Socs3 (Fig. 3C). GO analysis of differential genes indicated that SKF96365 participated in various biological, cellular, and molecular functions during SNL. GO analysis revealed the inhibition of SOCE induced long‐term biological changes in transcription, oxidation–reduction, signal transduction, and protein phosphorylation (Fig. 3D). Cellular functions were associated with alterations in the binding of ER and cell membrane including STIM1 and Orai1 tethering (Fig. 3D). The results also revealed specific enrichments in protein binding, molecular functions, ion or nucleotide binding, and ATP binding (Fig. 3D). In summary, transcriptome analyses found that SKF96365 enhanced gene expression by affecting multiple biological, cellular, and molecular activities.

### KEGG analysis revealed novel pathways involved in SOCE‐related interventions

KEGG analysis was used to identify more specific functional features before and after SOCE treatments. A total of 444 of 32 623 detected genes were significantly changed after nerve injury (Fig. S1a,b). KEGG identified several pathways involved in pain, including immune and inflammatory‐related responses, which is consistent with the findings of a prior study [35]. There was enrichment in pathways involved in response to mechanical stimulus, the extracellular space, lipopolysaccharide response, the extracellular region, and cytokine activity after the administration of SKF96365 (Fig. S1c,d). These results together suggested that SKF96365 may regulate neuronal response including cytokine activity, response to mechanical stimulus, and response to inflammatory mediators during neuropathic pain.

### Identification of SOCE‐related functional modules involved in neuropathic pain

WGCNA provides a reliable algorithm that constructs a bridge between clinical features, including paw withdrawal latency, following different treatments and gene expression in the spinal cord. This analysis is used to classify similar gene expression patterns, and a clustering system tree characterized by a co‐expression network (Fig. S2a). Using Module Eigengene (ME) analysis, the detected genes in Table S1 were divided into 15 modules and the correlations between each module were calculated by the correlative rate (Fig. S2b). Five clinical traits, including individual paw withdrawal latency, ligation, pain, injury, and anxiety, were recruited for further analysis. Since individual paw withdrawal latency provides more detail, the correlation between this feature and each ME was determined using the bicor and *P*‐value () functions. There is a total of phenotypes included in our study including paw withdrawal latency, ligation, pain, injury, and anxiety. We mainly focus on SKF96365‐induced analgesic effects, which had a relative higher relationship with paw withdrawal latency, as shown in Fig. S2c and Table 1.

**Table 1:** Pearson correlation analysis of modules with individual characters.

PWL	Ligation	Pain	Injury	Anxiety	
MEyellow	−0.2931	0.701533	−0.17998	0.701533	−0.17998
MEpurple	−0.48093	0.738688	0.189691	0.738688	0.189691
MEtan	−0.34073	0.6143	0.083687	0.6143	0.083687
MEbrown	0.270572	−0.09645	−0.1537	−0.09645	−0.1537
MEblack	−0.20452	0.326027	0.109734	0.326027	0.109734
MEred	0.345577	−0.71681	0.128808	−0.71681	0.128808
MEmagenta	0.568141	−0.63431	−0.20992	−0.63431	−0.20992
MEgreenyellow	0.014912	0.265889	−0.24299	0.265889	−0.24299
MEgreen	−0.02122	−0.22281	0.302131	−0.22281	0.302131
MEpink	0.02947	−0.02187	0.143998	−0.02187	0.143998
MEsalmon	−0.91431	0.799527	0.728945	0.799527	0.728945
MEcyan	−0.51683	0.531675	0.372346	0.531675	0.372346
MEmidnightblue	−0.3627	−0.09558	0.731599	−0.09558	0.731599
MEturquoise	−0.39324	0.000274	0.564433	0.000274	0.564433
MEblue	−0.49961	0.138336	0.519605	0.138336	0.519605
MEgrey	0.146389	0.045426	−0.23311	0.045426	−0.23311

### Functions and pathways involved in MEsalmon module

**Fig. 4:**
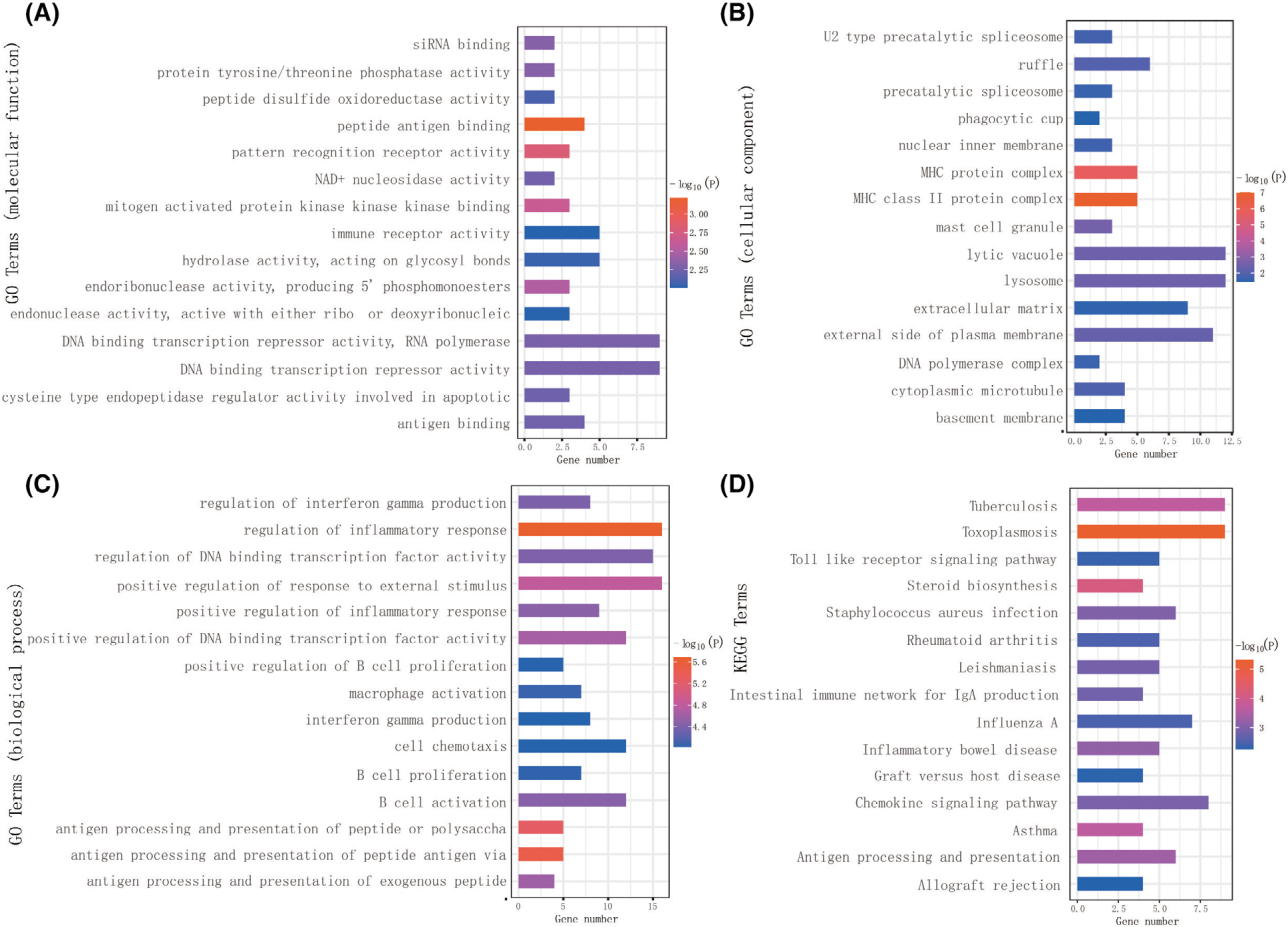
Functional and pathway enrichment analysis. Molecular process (A), cellular process (B), and biological process (C), and KEGG analysis (D) in MEsalmon module.

The signaling pathway that correlated most highly with the behavioral phenotype in SOCE‐mediated anti‐hypersensitivity was assessed. We found the MEsalmon module correlated most highly with paw withdrawal latency (Fig. S2c). Thus, GO function and KEGG pathway enrichment analyses were conducted to MEsalmon module. The results showed that MEsalmon module was involved in molecular processes such as transcription repression, immune receptor activity, hydrolase activity, and peptide antigen binding (Fig. 4A). From a cellular standpoint, the module was primarily associated with the external side of the plasma membrane, lysosome, lytic vacuole, extracellular matrix, and ruffle (Fig. 4B). The biological processes such as the regulation of inflammation, positive regulation of the response to external stimuli, and positive regulation of B cell proliferation, chemotaxis, and activation, were active (Fig. 4C). Fifteen KEGG pathways, including Toll‐like receptor signaling, steroid biosynthesis, and chemokine signaling, which may be involved in SOCE‐mediated pathways of neuropathic pain, were also identified (Fig. 4D). Therefore, these findings supported that Toll‐like receptor signaling, steroid biosynthesis, and chemokine signaling are crucial for SOCE‐mediated calcium influx and neuronal activity in neuropathic pain.

### SOCE inhibitor YM‐58483 inhibited neuropathic pain

**Fig. 5:**
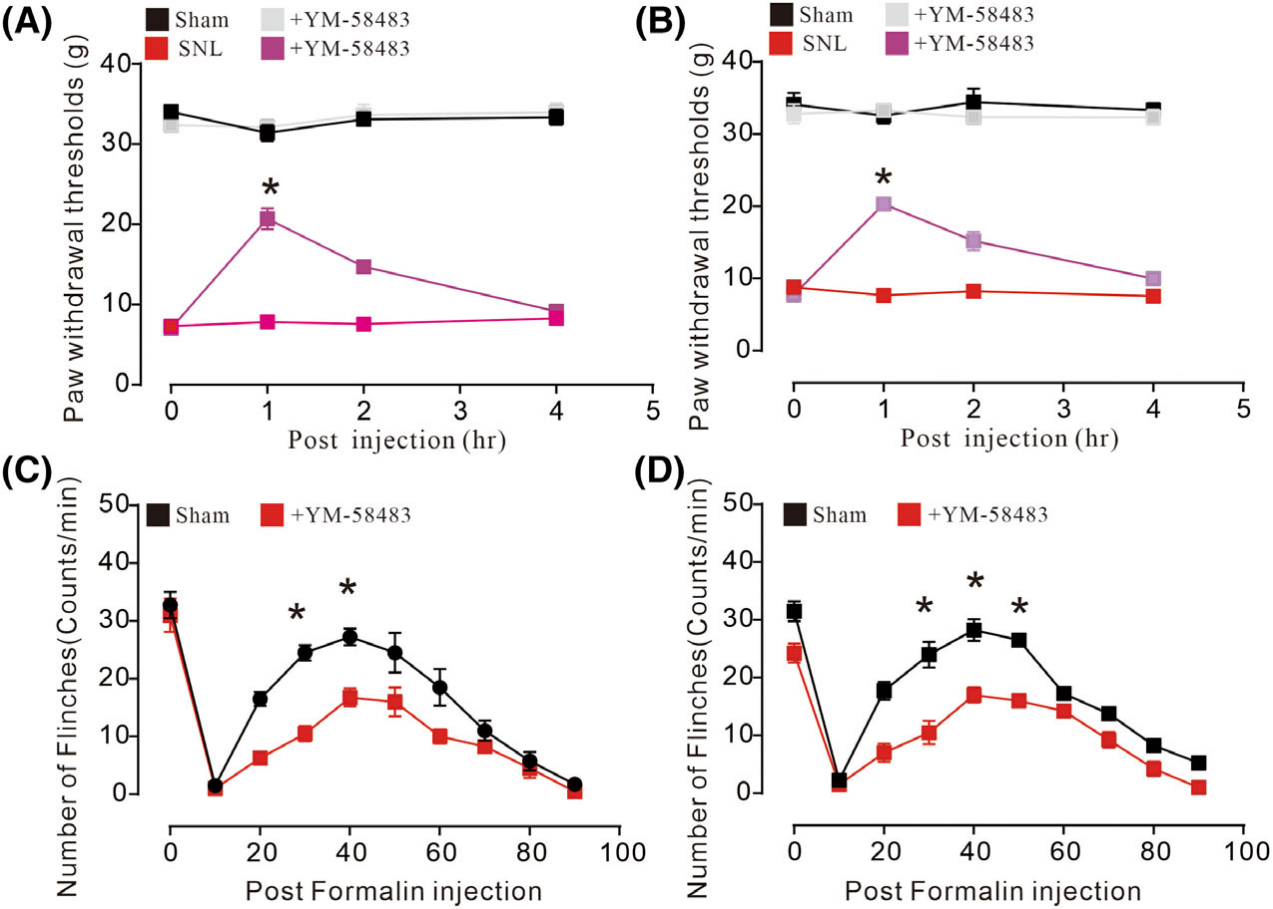
YM‐58483 suppressed pain states in male and female rats. YM‐58483 inhibited neuropathic [A, B, (A), *F*_(9,64)_ = 16.58, *P* < 0.0001, *P < 0.05, (B), *F*_(9,64)_ = 8.724, *P* < 0.0001, *P < 0.05, two‐way ANOVA followed by Tukey's post‐tests, *n* = 5] and formalin [C, D, (C), *F*_(9,60)_ = 5.1, *P* < 0.0001, *P < 0.05, (D), *F*_(9,60)_ = 3.772, *P* < 0.0001, *P < 0.05, two‐way ANOVA followed by Tukey's post‐tests, *n* = 4] pain in male and female rats. Data are represented as mean ± SEM.

In order to further demonstrate SOCE could be a promising target for pain relief, another inhibitor YM‐58483 was used to assess its analgesic effects. YM‐58483 was another potent inhibitor by suppressing SOCE‐mediated pain states. Orally delivery of YM‐58483 significantly suppressed nerve injury‐induced hypersensitivity in both male and female neuropathic rats. It remarkably prolonged mechanical thresholds to 20.69 g in male and 20.32 g in female rats [Fig. 5A,B, (A), *F*_(9,64)_ = 16.58, *P* < 0.0001, *P < 0.05, (B), *F*_(9,64)_ = 8.724, *P* < 0.0001, *P < 0.05, two‐way ANOVA followed by Tukey's post‐tests, *n* = 5]. Furthermore, YM‐58483 exerted inhibitory effects in formalin‐induced tonic pain in both male and female rats, which totally inhibited formalin‐induced flinches to 16.75 counts in male and 17 counts in female rats [Fig. 5C,D, (C), *F*_(9,60)_ = 5.1, *P* < 0.0001, (B), *F*_(9,60)_ = 3.772, *P* < 0.0001, *P < 0.05, two‐way ANOVA followed by Tukey's post‐tests, *n* = 4]. Therefore, inhibition of SOCE could be a promising target for pain relief.

## Discussion

Toll‐like receptors (TLRs), chemokine receptors, and purinoceptors (e.g., P2X4 receptors) on microglia rapidly responded to initiate signal transduction, expressing and releasing pro‐inflammatory factors such as TNF‐α, IL‐1β, IL‐6, and BDNF, reactive oxygen species, free radicals, and cytotoxic substances, thereby altering the central nervous system microenvironment and exacerbating the onset and progression of neurological diseases [35]. Similarly, activated microglia can release neuroinflammatory factors such as TNF‐α, IL‐1β, IL‐6, and BDNF, which enhance spinal dorsal horn neuronal firing through the presynaptic and postsynaptic membranes and cause pain in peripheral nerve damage. Human study has showed that the patients experiencing pain sensations have higher levels of IL‐1, IL‐6, and TNF‐α, which are correlated with the occurrence and prognosis of pain [36]. It has been demonstrated that bath application of external inflammatory mediator exerted long‐lasting and profound alterations in neuronal plasticity and behavioral phenotypes by promoting glutamatergic or inhibitory transmission, along with aberrant calcium capacity in synapse [37]. Early inflammatory cytokine productions resulted in multiple signaling abnormalities in GPCRs and channel activity [38]. Exposure to inflammatory mediators, serving as an early risk in pain, resulted in sensational impairments and led to gene habitations by penetrating synaptic clefts. Sustained production of inflammatory mediators could remarkably sensitize calcium dynamics through regulating GPCRs activity in synapse, promoting nocifensive reflexes during neuropathic pain. Calcium intensity is increasing recognized as a critical reason for neural plasticity and damage, exerting unparallel susceptibility following impairments in nocifensive transmissions. Calcium (Ca^2+^) channels are vital for neuronal functions, neurotransmitter release, synaptic plasticity, gene transcription, and behavioral alterations. They amplified incoming information through complex circuit interactions [39]. Interactions between STIM1 and Orai1 that mediate Ca^2+^ refilling are critical for maintaining SOCE when cellular Ca^2+^ levels are depleted. Through disrupting STIM1‐Orai1 interactions in the endoplasmic reticulum–plasma membrane pathway, both SKF96365 and YM‐58483 suppress calcium‐mediated transmitter release and neuronal activity while reducing hypersensitivity in neuropathic rats. The current study found that nerve injury promoted more calcium refilling than the sham group, and treatment with both SKF96365 and YM‐58483 caused a similar degree of antinociception by alleviating calcium homeostasis [40].

Nerve injury might sensitize Ca^2+^ channel in the presynaptic zone, which remolding neuronal activity in response to external stimulus [41]. Whole‐cell recording demonstrated that STIM1‐Orai1‐mediated calcium levels were enhanced after nerve injury as well as alterations of spinal glutamatergic transmission [42]. As a dominant ionic channel involved in maintaining synaptic homeostasis, alterations in presynaptic Ca^2+^ may increase the risk of disease later in life [43]. Several studies have shown that Ca^2+^ channel activation correlates positively with both neuronal excitability and hypersensitivity [44]. Pharmacological interventions by inhibiting Ca^2+^ channel with drugs such as ethosuximide have achieved great potential in dealing with nocifensive behaviors [45]. Previous studies show that SKF96365 and YM‐58483 possess the similar antiallodynic effects. This study provided further evidence that inhibition of SOCE by these two drugs had an analgesic effect via manipulating neuronal activity in the spinal dorsal horn. In addition, SKF96365 strongly suppressed the neuronal c‐Fos expression in neuropathic rats following mechanical stimuli, which further demonstrated that SOCE involved in neuronal pain circuits.

Sensory functions are dependent on spinal glutamatergic neurotransmission, which serves to mediate and respond to sensory information from the skin and deeper tissues. SOCE‐induced Ca^2+^ influx, as a critical channel mediating neuronal excitability and sensory responses, also potentiates cellular stabilization by altering glutamate receptors and neurotransmitter production [46]. The imbalance of glutamatergic and inhibitory transmission is also shown to affect the excitability of spinal cord neurons, cause aberrant receptor expression, and induce underlying molecular changes [29]. Consistent with the findings of this study, nerve injury has a long‐lasting effect on neurotransmitter release, indicating that alterations in neuroplasticity lead to behavioral sensitization in rats. Neuroplasticity is associated with a wide range of psychiatric disorders, including stroke, neurodegeneration, and pain, exerts profound effects on the regulation of compensatory and nocifensive behaviors [47]. Electrophysiological studies in rodents have shown that enhanced excitation after nerve injury may induce synapse remodeling, a process involving the stimulation of numerous synaptic arrangements that activates their postsynaptic targets and induces the release of cytokines [48]. Attached neurons from the spinal lamina II exhibit remarkable excitatory neurotransmitter release in response to excessive miniature excitatory postsynaptic currents (mEPSCs) associated with neuropathy [49], and produce mechanical hypersensitivity [50]. Indeed, the pharmacological inhibition of SOCE could significantly suppress nociceptive allodynia and induce mechanical hypersensitivity. The results shown that ablation of SOCE may also regulate Ca^2+^ in resting neurons and cause depolarization by binding with AMPA or NMDA in the postsynaptic zone. Thus, SOCE may be a promising target for manipulating neuronal activity by alleviating neuronal activity.

Molecular alterations are recognized as the basis for behavioral phenotypes and neural plasticity, likely contributing to nocifensive sensitization associated with neuropathic pain [51]. Proinflammatory factors such as NALP3 inflammasome, IL‐1β, and IL‐18, are shown to exhibit direct neural excitotoxicity that results in the accumulation of glutamate in the synaptic cleft and increases excitability at the early stage of pain [52]. Bath application of proinflammatory factors immediately enhances the frequency of spontaneous EPSCs (sEPSCs), which may further trigger long‐term synaptic plasticity by inducing inflammation [53]. The use of biomarkers to investigate proinflammatory factor production has gained increasing attention as a method for improving disease diagnosis [54]. Meanwhile, the use of non‐steroidal anti‐inflammatory drugs (NSAIDs) for inflammatory pain has provided a pharmacological basis for drug discovery since it has significant analgesic effects during experimental models of neuropathic and chronic pain [55]. In the current study, nerve injury altered gene expression and led to cellular impairments in transcription that changed responses to lipopolysaccharide, extracellular regions, and cytokine production. Our previous study also demonstrated that exenatide exhibited neuroprotective effects by remolding the transcriptional expression of inflammatory mediators during neuropathic pain [29]. Thus, the manipulation of spinal inflammation may allow for a more accurate assessment of compounds and the prediction of treatment outcomes.

The STIM1‐Orai1 interaction is necessary for SOCE‐mediated Ca^2+^ refilling. Using the STIM1 antagonist, SKF96365 and YM‐58483, this study identified a functional role for SOCE in pain relief. WGCNA showed that several molecular, cellular, and biological processes involved in pain relief were mediated by the inhibition of SOCE. It is well known that gene abnormalities in the spinal dorsal horn regulate kinase activity, protein conformation, channel homeostasis, and neural plasticity, thereby contributing to behavioral sensitization [56]. SKF96365 and YM‐58483 impede STIM1 and Orai1 binding and subsequently abolish fusion of the ER and plasma membrane. Bioinformatics analysis showed that SKF96365 augmented membrane activity, including the precatalytic spliceosome, nuclear inner membrane, lysosome, external side of the plasma membrane, cytoplasmic microtubules, and basement membrane, against nerve injury. Although our study demonstrated that targeting calcium channels helps to suppress neuropathic pain, further experiments including viral manipulation of calcium ion activity and *in vivo* recording may provide the causal evidence, be essential for a deeper understanding of the significance of SOCE.

Epigenetic and transcriptional alterations were reported that activator of nuclear receptor family 4a (NR4a) receptor, belongs to larger nuclear receptors superfamily of eukaryotic transcription factors, is participated in a variety of biological process including anti‐inflammatory capacity, memory formation, and neuroprotection as mediators of CREB experiencing pathological activators [57]. An animal study has demonstrated that a dramatic increase of NR4a, which may result in inflammatory activations, which contribute to the hypersensitivity in mice that are suffering chronic water‐avoidance stress [58]. Therefore, its unique sensitivity and epigenetic resilence in mediating anti‐inflammatory response is crucial for promoting TNF‐a‐induced pain states.

Therefore, nerve injury associated inflammatory factors promoted microglial activations and neuronal transmission by sensitized SOCE and calcium influx in synapse, resulted in activation of nocifensive reflexes in spinal cord. SKF96365 could remarkably suppressed SOCE‐mediated calcium influx and neuronal transmitter release, which subsequently alleviated cellular signaling including Toll‐like receptor signaling, steroid biosynthesis, chemokine signaling, and c‐Fos expressions, and glutamatergic transmission in neuropathic rats.

## Conflict of interest

The authors declare no conflict of interest.

### Peer review

The peer review history for this article is available at https://www.webofscience.com/api/gateway/wos/peer‐review/10.1002/2211‐5463.13699.

## Author contributions

LM, WW, YM, and JC conceived and designed the experiments. LM, QW, JH, HL, FL, XL, RL, and MX performed the experiments. QW and JH analyzed the data. LM and WW preparation of the manuscript. All authors contributed to the article and approved the submitted version.

## Supporting information


**Fig. S1.** Synergistic expression of the differential genes of pain following SKF96365 interventions. Heatmap (a) of z‐score transformed normalized expression values and KEGG analysis (b) for DEGs (SNL vs. Sham). Heatmap (c) of z‐score transformed normalized expression values and KEGG analysis (d) for DEGs (SKF96365 vs. SNL).

**Fig. S2.** Weighted gene co‐expression network analysis (WGCNA). The co‐expression groups via clustering were identified as modules (a). Heatmap of WGCNA module‐to‐module correlation (b). Heatmap of the correlation between different module eigengene and sample phenotype (c).

**Table S1.** Relevant information about detected genes in different modules.

## Data Availability

The raw data in our study are available from the corresponding author on reasonable requests.
