# Supplementary figures and images for: Store‐operated calcium entry mediates hyperalgesic responses during neuropathy
Source: FEBS Open Bio. 2023 Aug 28;13(11):2020–34. doi: 10.1002/2211-5463.13699 (PMC10626277; doi:10.1002/2211-5463.13699)

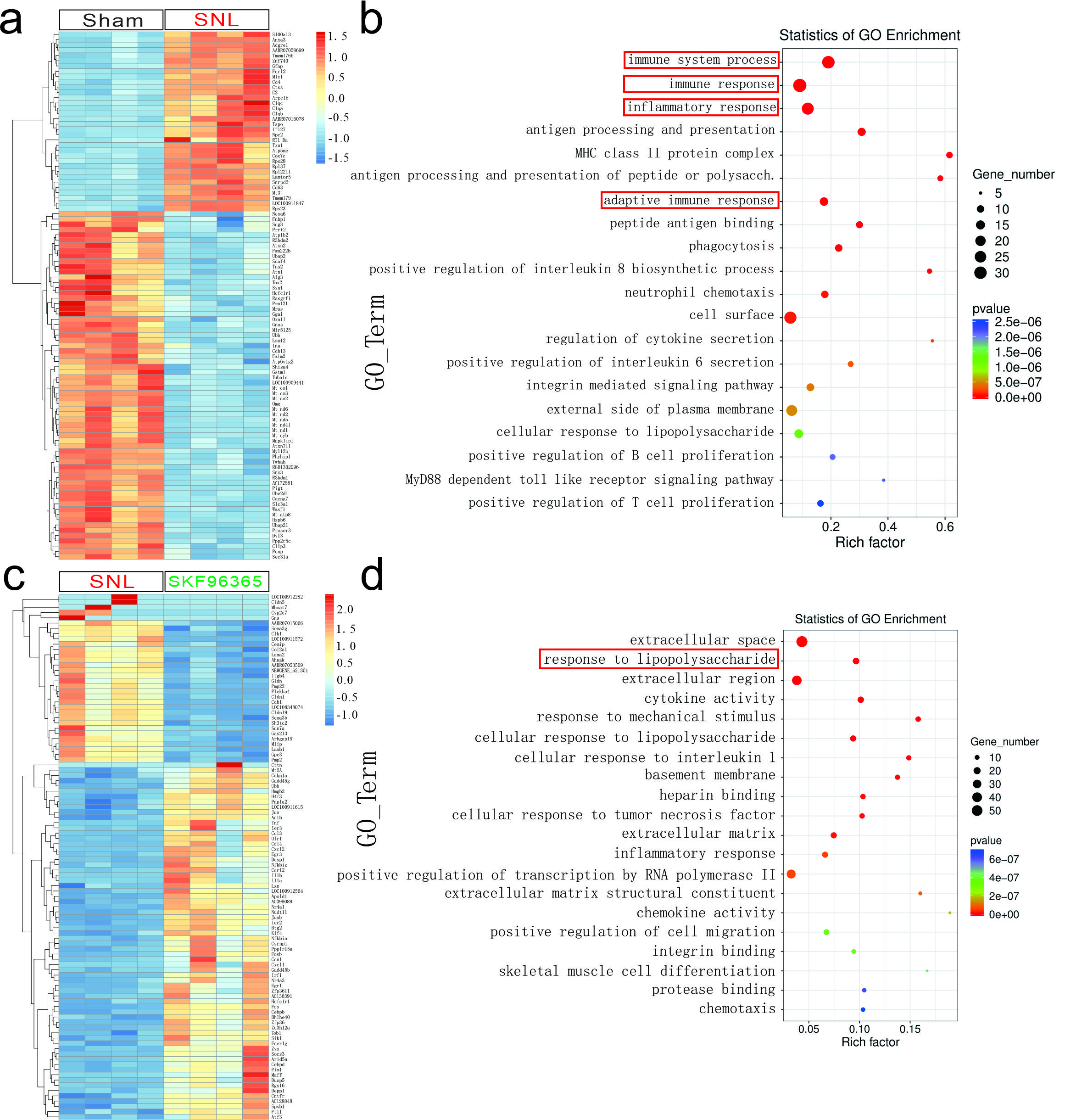

Supplement: Supplementary file 1 — Fig. S1. Synergistic expression of the differential genes of pain following SKF96365 interventions. Heatmap (a) of z‐score transformed normalized expression values and KEGG analysis (b) for DEGs (SNL vs. Sham). Heatmap (c) of z‐score transformed normalized expression values and KEGG analysis (d) for DEGs (SKF96365 vs. SNL). [file FEB4-13-2020-s001.jpg]

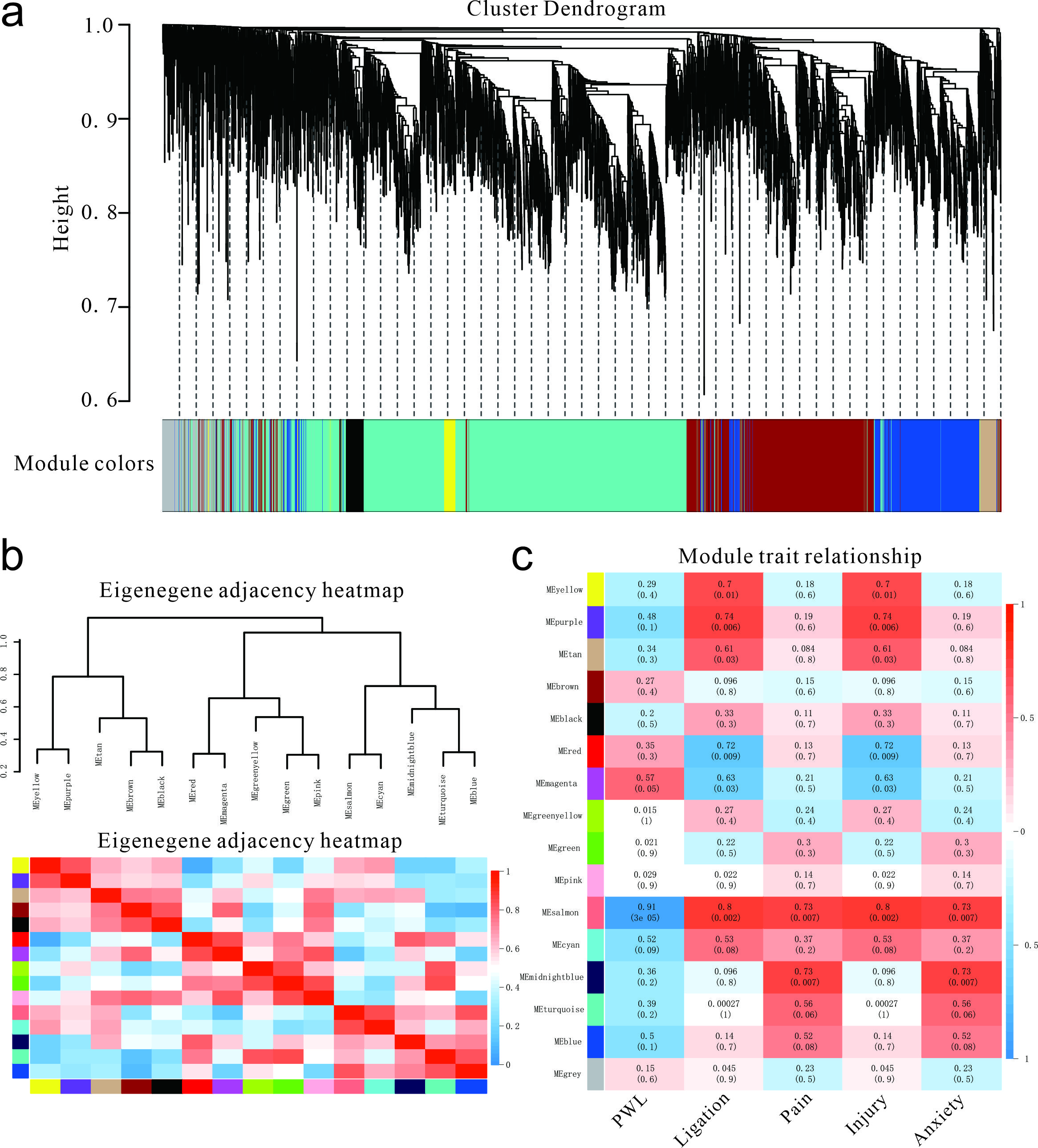

Supplement: Supplementary file 2 — Fig. S2. Weighted gene co‐expression network analysis (WGCNA). The co‐expression groups via clustering were identified as modules (a). Heatmap of WGCNA module‐to‐module correlation (b). Heatmap of the correlation between different module eigengene and sample phenotype (c). [file FEB4-13-2020-s002.jpg]
